# Living Alone as a Persistent Risk Factor for Smoking and High-Risk Drinking: A Three-Year Repeated Cross-Sectional Analysis Before and During the COVID-19 Pandemic in the Republic of Korea

**DOI:** 10.3390/healthcare14091251

**Published:** 2026-05-06

**Authors:** Sarang Jang

**Affiliations:** Department of Public Health, Sahmyook University, Seoul 01795, Republic of Korea; srjang@syu.ac.kr

**Keywords:** single-person household, smoking, high-risk drinking, COVID-19, Republic of Korea

## Abstract

**Background**: Single-person households have grown rapidly in the Republic of Korea and are consistently associated with higher rates of smoking and high-risk drinking. However, it remains unclear whether this vulnerability is structural and chronic or merely situational. This study examined whether the COVID-19 pandemic—a major societal disruption—altered the preexisting behavioral gap between single- and multi-person households. **Methods**: We used repeated cross-sectional data from the Korea Community Health Survey (KCHS) for 2019 (unweighted *n* = 229,099), 2020 (unweighted *n* = 229,269), and 2021 (unweighted *n* = 229,242), representing weighted populations of approximately 43.0, 43.5, and 43.6 million adults aged ≥19 years, respectively. We applied complex sample logistic regression models including an interaction term between survey year and household type, adjusting for sex, age, income, and education. **Results**: High-risk drinking significantly declined in both household types across all three time points following the COVID-19 pandemic, whereas smoking showed no significant overall change. Critically, the year x household type interaction was non-significant for high-risk drinking across all years; for smoking, a marginally significant interaction emerged only in 2021 (adjusted odds ratio [AOR] = 1.08, 95% CI: 1.01–1.16), suggesting a slight but limited divergence in the later pandemic period. Single-person households consistently showed higher odds of smoking (AOR = 1.65, 95% CI: 1.56–1.74) and high-risk drinking (AOR = 1.32, 95% CI: 1.25–1.39) across all three time points, relative to multi-person households and referenced to 2019 as the pre-pandemic baseline. **Conclusions**: The health behavioral vulnerability of single-person households is structural and persistent, underscoring the need for household-structure-sensitive public health strategies to promote sustainable well-being.

## 1. Introduction

### 1.1. Sustainable Well-Being and Structural Determinants of Health Behavior

Sustainable well-being encompasses not only the absence of disease but also the enduring capacity of individuals and communities to maintain health-promoting behaviors over time [1,2]. Among the behaviors most critical to this capacity, smoking and high-risk drinking are recognized as leading public health concerns, both of which are strongly associated with chronic disease burden and premature mortality [3,4] and are highly sensitive to social and environmental conditions [5,6]. Household composition, in particular, influences access to social support, behavioral norms, and informal regulation of daily routines, all of which are key mechanisms through which structural conditions sustain or undermine healthy behaviors over time [7]. Understanding the structural conditions that render certain populations persistently vulnerable to smoking and high-risk drinking and whether such vulnerability persists even under major societal disruptions is therefore essential for designing effective and sustainable public health interventions [8].

### 1.2. Single-Person Households and Health Behavioral Vulnerability

Single-person households have increased rapidly in the Republic of Korea, now representing one of the most prevalent household types in the country and exhibiting considerable heterogeneity in age, sex, and socioeconomic status [9,10,11]. Despite this diversity, individuals living alone consistently exhibit a higher prevalence of smoking and high-risk drinking than those in multi-person households [12,13]. The absence of co-residents who might provide social support, reinforce healthy routines, or exert informal behavioral control has been identified as a key mechanism underlying this vulnerability [12,14]. Living alone is also associated with elevated psychological distress, including depression and anxiety [14,15], as well as reduced regularity in diet and daily structure [14], all of which may further amplify the risk of smoking and high-risk drinking. Importantly, rather than treating single-person households as a homogeneous at-risk group, recent evidence highlights substantial internal heterogeneity within this population [16,17], suggesting that smoking and high-risk drinking patterns may differ significantly by age and sex. From a sustainable well-being perspective, this structural condition, namely the absence of the social and behavioral resources that co-residence provides, may weaken individuals’ capacity to maintain healthy behaviors, particularly under conditions of stress or societal disruption [18,19,20].

### 1.3. COVID-19 as a Natural Experiment

The COVID-19 pandemic created an unprecedented opportunity to examine whether single-person households’ vulnerability to smoking and high-risk drinking is structural or situational in nature. By imposing sudden and severe changes in social interaction, mobility, and everyday routines, the pandemic had the potential to either amplify existing disparities—by disproportionately increasing stress and isolation among those already living alone—or attenuate them—by reducing social drinking and smoking occasions for all household types equally [21,22]. Prior research has documented mixed findings regarding behavioral changes during the pandemic, with some studies reporting increases in smoking and alcohol use [22,23,24,25], and others reporting decreases [26,27,28,29], suggesting that the pandemic’s impact varied according to individual social circumstances and structural conditions [7,30]. Critically, however, most prior studies have focused on the direction of behavioral change at the population level or on simple comparisons between household types [13], with insufficient examination of whether the pandemic altered the relative gap in smoking and high-risk drinking between them. If single-person household vulnerability is primarily situational, it would be expected to fluctuate owing to a disruption of this magnitude. However, if the gap persists unchanged, this would constitute evidence consistent with a structural and chronic vulnerability, rooted in the enduring conditions of living alone, rather than in any particular external stressor [18].

### 1.4. Study Objectives

This study aimed to examine changes in smoking and high-risk drinking before and during the COVID-19 pandemic by household type using nationally representative data from the Republic of Korea, spanning three annual cross-sections (2019, 2020, and 2021), and to determine whether the pandemic altered the pre-existing disparity between single- and multi-person households over time. Subgroup analyses within single-person households were additionally conducted to explore heterogeneity by sex and age group. We hypothesized that: (1) single-person households would consistently show higher odds of both smoking and high-risk drinking across all three time points and (2) the interaction between survey year and household type would be non-significant, indicating that COVID-19 functioned as a stress test that revealed, rather than created or resolved, the structural and persistent nature of vulnerability to smoking and high-risk drinking among single-person households. The findings are discussed in the context of sustainable well-being and the social determinants of health behavior, with implications for public health policies targeting structurally vulnerable populations.

## 2. Materials and Methods

### 2.1. Study Design and Data Source

This study employed a repeated cross-sectional design using secondary data from the Korea Community Health Survey (KCHS) for 2019, 2020, and 2021. The KCHS is an annual nationally representative survey conducted by the Korea Disease Control and Prevention Agency (KDCA), targeting community-dwelling adults aged 19 years and older across all regions of the Republic of Korea. The survey is based on a complex two-stage sampling design. In the first stage, primary sampling units (dong/eup-myeon clusters) were selected using probability-proportional-to-size systematic sampling, stratified by administrative district and housing type, based on a sampling frame constructed from residential registration and housing records provided by the Ministry of the Interior and Safety and the Ministry of Land, Infrastructure and Transport. In the second stage, households within each selected cluster were chosen using systematic sampling, with an average of approximately 900 respondents targeted per public health center and a target margin of error of ±3%. Sampling weights were assigned to all respondents to account for the complex sampling design and ensure nationally representative estimates. The year 2019 was treated as the pre-COVID-19 reference period, 2020 as the early pandemic period during which large-scale social distancing measures and movement restrictions were implemented in the Republic of Korea, and 2021 as the ongoing pandemic period characterized by continued restrictions and the initial rollout of vaccination programs. Because the three annual surveys consisted of independent cross-sectional samples rather than repeated measurements of the same individuals, a repeated cross-sectional design was applied to compare group-level changes across time points. Therefore, a causal inference between the pandemic and health behavioral changes is not intended; rather, the study focuses on examining the associations and temporal trends.

### 2.2. Study Population

The unweighted sample comprised 229,099 adults in 2019 (multi-person households: *n* = 192,521; single-person households: *n* = 36,578), 229,269 adults in 2020 (multi-person households: *n* = 192,127; single-person households: *n* = 37,142), and 229,242 adults in 2021 (multi-person households: *n* = 189,285; single-person households: *n* = 39,957), representing weighted populations of approximately 43.0 million, 43.5 million, and 43.6 million, respectively. Single-person households were over-represented in the unweighted sample (approximately 17%) relative to their estimated population proportion (approximately 12–14%), reflecting the complex sampling design of the KCHS; all analyses incorporated sampling weights to account for this design effect. Participants were classified into two household types based on the number of residents within the household: single-person households (living alone) and multi-person households (two or more residents). Single-person households were defined as those who responded as living alone in the household composition item of the KCHS, and multi-person households were defined as all remaining cases.

### 2.3. Variables

#### 2.3.1. Outcome Variables

Two substance-related behaviors were examined as outcome variables: current smoking and high-risk drinking. Current smoking was defined as currently smoking, based on a single survey item asking whether the respondent currently smokes and was coded as a binary variable (0 = non-smoker, 1 = current smoker). High-risk drinking was defined as consuming 7 or more drinks per occasion for men (or 5 cans of beer equivalent) or 5 or more drinks per occasion for women (or 3 cans of beer equivalent) two or more times per week in the past year, in accordance with the standard KCHS definition [31] which has been consistently applied in prior studies examining alcohol-related health behaviors using KCHS data in the Republic of Korea [12,13] and was similarly coded as a binary variable (0 = non-high-risk drinker, 1 = high-risk drinker).

#### 2.3.2. Key Independent Variable

Three key independent variables were included in the analysis: survey year and household type, along with their interaction. The survey year was coded as a categorical variable with three levels (2019, 2020, and 2021), with 2019 designated as the reference category to reflect the pre-COVID-19 baseline. Household type was coded using multi-person households as the reference category (multi-person = 0, single-person = 1). Interaction terms between survey year and household type (2020 × single-person and 2021 × single-person) were constructed to assess whether the magnitude of the change in smoking and high-risk drinking across the three time points differed by household type.

#### 2.3.3. Covariates

To control for potential confounders, the following sociodemographic variables were included as covariates: sex (male, female), age group (19–29, 30–44, 45–64, ≥65 years), education level (elementary school or below, middle school, high school, college or above), and monthly household income level (<1,000,000 KRW, 1,000,000–1,990,000 KRW, 2,000,000–2,990,000 KRW, ≥3,000,000 KRW). Age groups were defined based on life-course stages and distinct health behavioral characteristics. All the covariates were treated as dummy variables in the regression model.

### 2.4. Statistical Analysis

The three annual KCHS datasets (2019, 2020, and 2021) were merged for the analysis. All analyses were conducted using complex sample analytical methods, incorporating sampling weights, stratification variables, and cluster variables to account for the complex sampling design of the KCHS, thereby ensuring nationally representative estimates.

General characteristics of the study population were described by household type and survey year using weighted frequencies and proportions. Differences between groups were assessed using the Rao–Scott chi-square test, which is appropriate for complex sample data.

To examine the changes in smoking and high-risk drinking before and during the COVID-19 pandemic, complex sample logistic regression models were separately fitted for each outcome. Each model included the survey year, household type, and their interaction term as the primary predictors, along with sex, age group, income level, and education level as covariates. The interaction term (year × household type) was the primary test used to determine whether COVID-19 differentially affected the two household types. The results are presented as adjusted odds ratios (AORs) with 95% confidence intervals (CIs).

Additionally, subgroup analyses were conducted within single-person households to explore heterogeneity in changes in smoking and high-risk drinking by sex and age group. All the regression models were adjusted for the same set of covariates.

All statistical analyses were performed using the IBM SPSS Statistics version 30 (IBM Corp., Armonk, NY, USA). Statistical significance was set at *p* < 0.05 (two-tailed).

### 2.5. Ethical Considerations

Ethical review and approval were waived for this study because it used publicly available and de-identified secondary data from the Korea Community Health Survey (KCHS), which does not involve direct human subject research.

## 3. Results

### 3.1. General Characteristics of the Study Population

General characteristics of the study population are summarized in Table 1. The proportion of single-person households increased progressively across the three time points (12.32% in 2019, 12.93% in 2020, and 14.29% in 2021; *p* < 0.001). The sex distribution remained stable across all years (males: approximately 49.56–49.58%; *p* = 0.992). However, the age distribution shifted significantly (*p* < 0.001), with a gradual decrease in younger age groups (19–29 and 30–44 years) and a progressive increase in the proportion of adults aged ≥65 years (19.07% in 2019 to 20.65% in 2021). Income distribution also changed significantly (*p* < 0.001), with the highest income category (≥3,000,000 KRW) declining from 67.32% in 2019 to 65.59% in 2021, and increases in lower income categories. Education levels shifted significantly (*p* < 0.001), with the proportion having a college degree or above increasing from 42.04% in 2019 to 44.12% in 2021.

### 3.2. Trends in Smoking and High-Risk Drinking by Household Type

**Figure 1:**
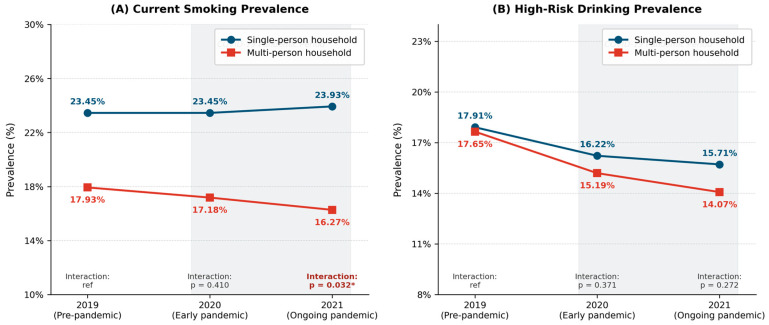
Weighted prevalence of current smoking (**A**) and high-risk drinking (**B**) by household type, 2019–2021. Notes: Interaction *p*-values are for the Year × Household Type term from complex sample logistic regression models. * *p* < 0.05.

Descriptive trends for smoking and high-risk drinking across the three time points are shown in Table 2. For smoking, notably different patterns emerged between the two household types. Among multi-person households, current smoking prevalence declined consistently across years, from 17.93% in 2019 to 17.18% in 2020 and 16.27% in 2021 (*p* < 0.001). Among single-person households, smoking prevalence showed no significant change across all three years (23.45% in 2019, 23.45% in 2020, and 23.93% in 2021; *p* = 0.511), suggesting a persistent plateau. Across all three time points, single-person households consistently exhibited higher smoking prevalence than multi-person households. For high-risk drinking, both household types showed progressive and significant declines over time. Among single-person households, high-risk drinking decreased from 17.91% in 2019 to 16.22% in 2020 and 15.71% in 2021 (*p* < 0.001). Among multi-person households, it declined from 17.65% in 2019 to 15.19% in 2020 and 14.07% in 2021 (*p* < 0.001). Additionally, both current smoking and high-risk drinking were consistently more prevalent among single-person households than multi-person households across all time points, indicating a persistent disparity in health behaviors between the two groups. Figure 1 illustrates these prevalence trends across the three time points by household type, visually demonstrating the largely parallel trajectories between single- and multi-person households for both outcomes.

### 3.3. Adjusted Associations of Household Type with Smoking and High-Risk Drinking

The results of the complex sample logistic regression analysis are presented in Table 3. For smoking, a significant overall year effect was observed: smoking odds declined progressively in 2020 (AOR = 0.95, 95% CI: 0.92–0.97, *p* < 0.001) and further in 2021 (AOR = 0.90, 95% CI: 0.88–0.93, *p* < 0.001) relative to 2019. For high-risk drinking, a significant and progressive decline was confirmed in 2020 (AOR = 0.83, 95% CI: 0.81–0.86, *p* < 0.001) and 2021 (AOR = 0.77, 95% CI: 0.74–0.79, *p* < 0.001) relative to 2019.

Regarding the interaction terms, the 2020 × single-person interaction was non-significant for both smoking (AOR = 1.03, 95% CI: 0.96–1.10, *p* = 0.410) and high-risk drinking (AOR = 1.03, 95% CI: 0.96–1.11, *p* = 0.371), suggesting that the early pandemic period did not differentially affect the two household types. For 2021, the smoking interaction term reached marginal significance (AOR = 1.08, 95% CI: 1.01–1.16, *p* = 0.032), suggesting that smoking declined somewhat less among single-person households relative to multi-person households in the later pandemic period; however, this effect was modest in magnitude. The 2021 × single-person interaction for high-risk drinking remained non-significant (AOR = 1.04, 95% CI: 0.97–1.11, *p* = 0.272), indicating that the progressive decline in high-risk drinking was uniform across household types throughout all three time points. Single-person households showed significantly higher odds of smoking (AOR = 1.65, 95% CI: 1.56–1.74, *p* < 0.001) and high-risk drinking (AOR = 1.32, 95% CI: 1.25–1.39, *p* < 0.001) compared to multi-person households across all years.

Regarding covariates, sex was the strongest predictor of both outcomes: males showed substantially higher odds of smoking (AOR = 17.11, 95% CI: 16.62–17.61) and high-risk drinking (AOR = 4.35, 95% CI: 4.26–4.44) compared to females (both *p* < 0.001). Younger age groups consistently showed higher odds of both outcomes than those aged 65 years or older. Lower income was associated with higher odds of smoking but lower odds of high-risk drinking, whereas lower education was associated with higher odds of both outcomes (all *p* < 0.001).

### 3.4. Subgroup Analysis Within Single-Person Households

The subgroup analyses within single-person households revealed notably different smoking and high-risk alcohol consumption patterns across all three time points (Table 4). For smoking, no significant overall change was observed in 2020 (AOR = 0.98, 95% CI: 0.93–1.04, *p* = 0.573) or in 2021 (AOR = 0.99, 95% CI: 0.93–1.05, *p* = 0.652) relative to 2019, consistent with the overall results. The age-stratified results were heterogeneous: adults aged 30–44 years showed a significant decrease in smoking odds in both 2020 (AOR = 0.85, 95% CI: 0.76–0.95, *p* = 0.005) and 2021 (AOR = 0.79, 95% CI: 0.71–0.88, *p* < 0.001), while those aged 45–64 years showed no significant changes. No significant changes were observed in the 19–29 or ≥65 age groups. Sex-stratified analyses indicated a significant decline among females in 2021 (AOR = 0.88, 95% CI: 0.78–0.98, *p* = 0.025) but not in 2020.

In contrast, high-risk drinking showed a consistent and significant decline across all subgroups for both 2020 and 2021 relative to 2019. The overall reductions were significant in both 2020 (AOR = 0.87, 95% CI: 0.82–0.93, *p* < 0.001) and 2021 (AOR = 0.81, 95% CI: 0.76–0.86, *p* < 0.001), and these patterns held for both males and females across all age groups.

## 4. Discussion

### 4.1. Persistent Vulnerability of Single-Person Households

The central finding of this study is not that COVID-19 changed smoking and high-risk drinking; it is that COVID-19 largely did not change the gap between household types. Across three annual time points spanning the pre-pandemic period and two years of the COVID-19 pandemic, the interaction between survey year and household type was non-significant in 2020 for both outcomes, and in 2021 for high-risk drinking, suggesting that the pandemic neither widened nor narrowed the pre-existing disparity between single- and multi-person households during these early-to-mid pandemic phases. The one notable exception was a marginally significant interaction in 2021 for smoking (AOR = 1.08, 95% CI: 1.01–1.16, *p* = 0.032), suggesting that smoking declined slightly less among single-person households than multi-person households in the later pandemic period; however, this effect was modest in magnitude and should be interpreted with caution, as discussed further in Section 4.3.

Across all three time points, single-person households consistently showed higher odds of smoking (AOR = 1.65, 95% CI: 1.56–1.74) and high-risk drinking (AOR = 1.32, 95% CI: 1.25–1.39) than multi-person households. Taken together, these findings are consistent with the interpretation that the vulnerability of single-person households to smoking and high-risk drinking is consistent with enduring structural conditions rather than situational factors alone. The pandemic functioned as a multi-year observational context in which the relative behavioral gap between household types could be examined across multiple phases of societal disruption. While the overall pattern of stability across three time points and two distinct behavioral outcomes lends some support to a structural interpretation, it is important to acknowledge that non-significant interaction terms do not constitute proof of equivalence; limited statistical power to detect small but meaningful differences in interaction effects cannot be ruled out [32,33]. Simulation studies have shown that interaction effects are generally small and difficult to detect, and that true moderation effects frequently go unidentified, leading to potential Type II errors [34]. Formally establishing equivalence would require dedicated analytical approaches—such as equivalence testing or Bayesian factor analysis—which were beyond the scope of the present study [35]. Alternative explanations—including insufficient magnitude or duration of disruption, or unmeasured confounding—remain plausible and should temper overly strong causal conclusions [36].

Nevertheless, this finding extends prior research documenting a higher prevalence of smoking and high-risk drinking among single-person households [19,20], by suggesting that this disparity may persist across markedly different social environments [18,19]. The consistency of the pattern across two pandemic years and two behavioral outcomes provides a basis for ongoing investigation into the structural mechanisms underlying this vulnerability.

### 4.2. High-Risk Drinking: A Socially Driven Behavior

The progressive and significant overall decline in high-risk drinking observed across 2020 and 2021 in both household types is consistent with prior research documenting reductions in alcohol consumption during the pandemic [37,38,39,40,41]. This pattern likely reflects the disruption of social drinking occasions, including in gatherings, bars, and parties, due to social distancing measures and movement restrictions [37,39,42]. The sustained uniformity of this decline across household types across both 2020 and 2021, reflected in consistently non-significant interaction terms for high-risk drinking, aligns with evidence that living arrangements and co-residence status are not consistently associated with changes in drinking behavior during the pandemic [43,44] and supports the interpretation that the reduction was primarily driven by changes in the social environment rather than by household-level factors. However, it is important to note that the overall decline does not imply reduced risk for all individuals. Prior research has shown that groups experiencing psychological and economic stressors, including job loss, income reduction, and elevated depression and anxiety, showed increases in alcohol use during the pandemic [39,42]. The average decline observed in this study may therefore mask heterogeneous patterns within subgroups, particularly among those with pre-existing high-risk drinking or severe psychosocial stressors [38,45,46,47]. Nonetheless, the consistent decline across all age groups and both sexes within single-person households observed in the subgroup analyses suggests that the social-environmental effect of COVID-19 on high-risk drinking was robust within this population.

### 4.3. Smoking: Habit-Driven and Structurally Embedded, and Age-Differentiated

In contrast to high-risk drinking, smoking showed no significant overall trend of change following COVID-19 at the population level, and the year × household type interaction was non-significant in 2020 for both household types. This divergence reflects a fundamental difference in the behavioral nature of the two outcomes: while drinking is more heavily influenced by social context and opportunity, smoking is more strongly governed by individual habits and nicotine dependence, rendering it comparatively less responsive to situational changes in the social environment [48,49].

However, a marginally significant interaction emerged in 2021 (AOR = 1.08, 95% CI: 1.01–1.16, *p* = 0.032), indicating that as the pandemic extended into its second year, the modest overall decline in smoking was concentrated more heavily in multi-person households. This pattern may reflect the cumulative effect of sustained health-risk awareness and social reinforcement operating within co-residential settings over time—mechanisms that may be less accessible to those living alone [20,50]. Notably, this divergence was modest in magnitude and should be interpreted with caution; it does not overturn the broader finding of relative stability, but rather suggests that the social reinforcement advantage of co-residence may become more pronounced over time under prolonged societal stress [51,52].

Of particular note is the finding that smoking did not decline among single-person households even in the context of COVID-19, a respiratory infectious disease that heightened public awareness of the health risks of smoking [53,54,55]. While multi-person households showed a modest but statistically significant decrease in smoking prevalence across the three time points, single-person households showed no such change—and the descriptive data even suggest a slight increase by 2021 (23.45% in 2019 and 2020 to 23.93% in 2021). This pattern may indicate that single-person households may be less able to translate heightened health-risk awareness into behavioral change, potentially due to the absence of social reinforcement and co-resident support that are known to facilitate smoking cessation [20,50,53].

The subgroup analyses within single-person households revealed notable age-differentiated patterns in smoking that warrant deeper interpretation. Adults aged 30–44 years showed a significant and progressive decline in smoking odds in both 2020 (AOR = 0.85, *p* = 0.005) and 2021 (AOR = 0.79, *p* < 0.001) relative to 2019, whereas those aged 45–64 years showed no significant change in either year—and the direction of the estimates even suggested a slight, non-significant increase. These divergent patterns may reflect differences in the social and occupational contexts of these age groups during the pandemic.

For adults aged 30–44 years, the pandemic period was associated with widespread shifts toward remote work arrangements, which may have reduced workplace smoking opportunities and peer smoking cues [50,53,56]. Additionally, this age group is more likely to include individuals with dependent family members—such as young children or aging parents—whose presence at home during lockdowns may have introduced informal social accountability that discouraged smoking [20,55]. Heightened sensitivity to COVID-19-related health risks may also have been more salient in this group, as they may have perceived themselves as both personally vulnerable and responsible for protecting household members [57].

In contrast, adults aged 45–64 years within single-person households showed no significant reduction in smoking. This age group is more likely to include long-term, heavily dependent smokers for whom nicotine dependence is more entrenched, reducing the responsiveness to situational cues or motivational changes [48,49]. Furthermore, this group may have faced elevated occupational stress and economic uncertainty during the pandemic, factors that are known to sustain or increase smoking behavior [24,25,54,58]. The absence of co-resident social support, which is particularly critical for smoking cessation in older adults [50,59,60], may have further limited their ability to translate health-risk awareness. Taken together, these findings suggest that patterns consistent with structural vulnerability may vary across life stages, underscoring the need for age-sensitive cessation interventions within this population [61,62].

### 4.4. Interpreting the Discrepancy Between Prevalence Rates and Adjusted Odds Ratios for High-Risk Drinking

A finding that may initially appear counterintuitive is the discrepancy between the raw prevalence of high-risk drinking and the adjusted odds ratios across household types. Descriptively, the difference in high-risk drinking prevalence between single-person households (17.91% in 2019) and multi-person households (17.65% in 2019) appears modest. Yet the adjusted odds ratio for single-person households was notably elevated (AOR = 1.32, 95% CI: 1.25–1.39). This discrepancy is not a statistical artifact but reflects the logic of covariate adjustment in multivariate regression [63].

The complex sample logistic regression models adjust for sex, age group, income level, and education—all of which differ systematically between household types and are independently associated with high-risk drinking. Compared to multi-person households, single-person households in the Republic of Korea are disproportionately composed of older adults, individuals with lower incomes, and those with lower educational attainment [9,10,11], all of which are sociodemographic profiles associated with lower odds of high-risk drinking in the adjusted model. In other words, based solely on their demographic composition, single-person households would be expected to show lower rates of high-risk drinking than multi-person households. The fact that they nonetheless exhibit comparable or higher raw prevalence—and substantially higher adjusted odds—suggests that living alone is associated with a higher adjusted risk after accounting for demographic factors [12,13].

The adjusted odds ratio thus captures the independent effect of household type after accounting for sociodemographic factors, and the two should not be interpreted as contradictory but as complementary perspectives on the same underlying phenomenon [63,64].

### 4.5. Implications for Sustainable Well-Being Policy

Interpreted through the lens of sustainable well-being, the consistent vulnerability of single-person households to smoking and high-risk drinking, unchanged even by a major societal disruption, points to a fundamental deficit in the structural resources needed to sustain healthy behaviors over time. Co-residence provides a range of behavioral and social buffers, including shared daily routines, informal monitoring of health behaviors, emotional support, and social accountability, all of which may facilitate the long-term maintenance of healthy behaviors [20]. In their absence, single-person households may lack the social infrastructure that underpins sustainable well-being, leaving them more exposed to smoking and high-risk drinking, regardless of external circumstances. This structural interpretation has direct implications for public health policies. Interventions targeting only situational stressors, such as crisis-specific responses to pandemics, are insufficient for this population. What is needed are sustained, structural approaches that address the social conditions of living alone: community-based social support programs, peer networks, and household-sensitive health promotion strategies that can compensate for the behavioral buffers provided by co-residence [65,66]. Furthermore, the internal heterogeneity within single-person households observed in the subgroup analyses, particularly the age-differentiated patterns in smoking, underscores the need for targeted interventions that account for variations within this population, rather than treating single-person households as a homogeneous group [16,17,67,68,69].

### 4.6. Strengths and Limitations

This study has several strengths. First, the use of nationally representative data from the KCHS with complex sample analytical methods supports the generalizability of the findings to the the Republic of Korean adult population. Second, the repeated cross-sectional design spanning three annual time points (2019, 2020, and 2021)—covering the pre-pandemic baseline, the early pandemic period, and the ongoing pandemic period—combined with the inclusion of interaction terms between year and household type, enabled examination of whether COVID-19 differentially affected the two household types across multiple phases of the pandemic. Third, the simultaneous examination of two distinct substance-related behaviors allowed for a comparative understanding of how the pandemic’s effects may differ by behavioral type across time.

However, several limitations must be acknowledged. First, the repeated cross-sectional design precludes tracking individual-level changes over time or establishing causal relationships between household type and health behavior. Second, both outcome variables were based on self-reported data, which may have been subject to social desirability bias and measurement error. Additionally, the frequency-based definition of high-risk drinking used in this study may not fully capture context-specific changes in drinking patterns during the pandemic, such as shifts from social to solitary drinking occasions, which may have varied systematically by household type [44,70]. Third, while the study covers three annual waves through 2021, the findings reflect the early-to-mid pandemic period only, and behavioral patterns may have continued to evolve in subsequent years, particularly with the easing of restrictions and the broader rollout of vaccination programs. Fourth, although key sociodemographic covariates were included, unmeasured confounders—such as mental health status, employment changes, and degree of social isolation, all of which were particularly relevant during the COVID-19 period—were not available in the dataset and may have influenced both the main effects and subgroup findings [46]. Fifth, and importantly, the non-significant interaction terms reported in this study should not be interpreted as evidence of equivalence or true stability between household types. Interaction effects are inherently more difficult to detect than main effects, and the present study may have had limited statistical power to identify small but potentially meaningful differences in behavioral trajectories across household types [71]. The observed pattern of stability is therefore best understood as consistent with, rather than definitive proof of, a structural interpretation. Future studies should incorporate longitudinal designs [72], a broader range of psychosocial factors [73], and equivalence testing frameworks to more rigorously examine how household composition shapes smoking and high-risk drinking over time [74].

## 5. Conclusions

This study examined changes in smoking and high-risk drinking before and during the COVID-19 pandemic by household type, using nationally representative data from the Republic of h Korea across three annual time points (2019, 2020, and 2021). The findings consistently point to one central conclusion: the vulnerability of single-person households to smoking and high-risk drinking is consistent with a structural and persistent pattern. Despite the profound and prolonged social disruption brought about by the COVID-19 pandemic, the disparity between single- and multi-person households remained largely unchanged; across both 2020 and 2021, the pandemic neither widened nor narrowed the pre-existing gap for high-risk drinking, and any deviation for smoking was modest in magnitude and limited to the later pandemic period. Single-person households showed consistently higher odds of both smoking and high-risk drinking across all three time points, suggesting that household composition functions as a meaningful and enduring structural determinant of these behaviors. It is important to note, however, that the non-significant interaction terms on which this interpretation relies do not constitute proof of equivalence, and alternative explanations cannot be ruled out.

The divergent patterns observed for smoking and high-risk drinking further illuminate the mechanisms at play. The overall decline in high-risk drinking across both household types reflects the disruption of social drinking opportunities during the pandemic, underscoring the socially driven nature of this behavior. In contrast, the absence of meaningful change in smoking, particularly the lack of decline among single-person households even amid heightened health-risk awareness, highlights the habit-driven and dependence-based nature of smoking and suggests that single-person households may be less equipped to translate health-risk awareness into behavioral change without the social reinforcement that co-residence provides. Notably, the age-differentiated smoking patterns observed within single-person households—with adults aged 30–44 years showing a significant decline and those aged 45–64 years showing no meaningful change—suggest that this structural vulnerability is not uniform but varies across life stages.

From the sustainable well-being perspective, these findings convey an important message: public health strategies that respond only to situational crises will not be sufficient to address the structural vulnerability of single-person households. As this population continues to grow in the Republic of Korea and globally, sustained and targeted interventions are needed—ones that address the enduring social conditions of living alone, compensate for the absence of co-resident behavioral support, and are sensitive to internal heterogeneity within single-person households across age and sex. Household composition should be recognized as a core social determinant of health behavior in public health policy, and the promotion of sustainable well-being among single-person households must be treated as a long-term structural priority, rather than a crisis-driven response.

## Figures and Tables

**Table 1:** General characteristics of the study population by year.

Variables	2019 (%)	2020 (%)	2021 (%)	*p*
Unweighted total (*n*)	229,099	229,269	229,242	
Single-person household	36,578	37,142	39,957	
Multi-person household	192,521	192,127	189,285	
Weighted population (*n*)	43,038,864	43,526,824	43,609,675	
Single-person household	5,303,521	5,628,775	6,231,058	
Multi-person household	37,735,343	37,898,049	37,378,617	
Household type				
Single-person household	12.32	12.93	14.29	<0.001
Multi-person household	87.68	87.07	85.71	
Sex				
Male	49.56	49.56	49.58	0.992
Female	50.44	50.44	50.42	
Age group (in years)				
19–29	17.29	17.00	16.63	<0.001
30–44	25.20	24.74	24.49	
45–64	38.44	38.47	38.22	
≥65	19.07	19.79	20.65	
Income level				
<1,000,000 KRW	8.05	9.33	9.02	<0.001
1,000,000–1,990,000 KRW	10.97	11.82	11.24	
2,000,000–2,990,000 KRW	13.65	14.52	14.15	
≥3,000,000 KRW	67.32	64.32	65.59	
Education level				
Elementary school or below	12.69	12.50	11.75	<0.001
Middle school	8.19	8.05	7.71	
High school	37.08	37.44	36.41	
College or above	42.04	42.01	44.12	

Note: *p*-values were calculated using the Rao–Scott chi-square test.

**Table 2:** Prevalence of smoking and high-risk drinking before and during COVID-19 by household type.

	Single-Person Household				Multi-Person Household			
Variables	2019 (%)	2020 (%)	2021 (%)	*p*	2019 (%)	2020 (%)	2021 (%)	*p*
Current smoking								
Non-smoker	76.55	76.55	76.07	0.511	82.07	82.82	83.73	<0.001
Current smoker	23.45	23.45	23.93		17.93	17.18	16.27	
High-risk drinking								
Non-high-risk drinker	82.09	83.78	84.29	<0.001	82.35	84.81	85.93	<0.001
High-risk drinker	17.91	16.22	15.71		17.65	15.19	14.07	

Note: *p*-values were calculated using the Rao–Scott chi-square test.

**Table 3:** Adjusted odds ratios for smoking and high-risk drinking with interaction between year and household type.

Variables	Smoking			High-Risk Drinking		
	AOR	95% CI	*p*	AOR	95% CI	*p*
Year						
2019 (Ref.)	-	-		-	-	
2020	0.95	0.92–0.97	<0.001	0.83	0.81–0.86	<0.001
2021	0.90	0.88–0.93	<0.001	0.77	0.74–0.79	<0.001
Household type						
Single-person household	1.65	1.56–1.74	<0.001	1.32	1.25–1.39	<0.001
Multi-person household (Ref.)	-	-		-	-	
Year × Household type						
2019 × Multi-person (Ref.)	-	-		-	-	
2020 × Single-person	1.03	0.96–1.10	0.410	1.03	0.96–1.11	0.371
2021 × Single-person	1.08	1.01–1.16	0.032	1.04	0.97–1.11	0.272
Sex						
Male	17.11	16.62–17.61	<0.001	4.35	4.26–4.44	<0.001
Female (Ref.)	-	-		-	-	
Age group						
19–29	2.70	2.59–2.82	<0.001	3.05	2.92–3.19	<0.001
30–44	4.47	4.30–4.64	<0.001	4.09	3.93–4.26	<0.001
45–64	3.59	3.48–3.71	<0.001	3.24	3.13–3.36	<0.001
≥65 (Ref.)	-	-		-	-	
Income level						
<1,000,000 KRW	1.23	1.18–1.28	<0.001	0.69	0.65–0.72	<0.001
1,000,000–1,990,000 KRW	1.21	1.17–1.25	<0.001	0.77	0.74–0.80	<0.001
2,000,000–2,990,000 KRW	1.21	1.18–1.25	<0.001	0.92	0.89–0.94	<0.001
≥3,000,000 KRW (Ref.)	-	-		-	-	
Education level						
Elementary school or below	1.85	1.77–1.93	<0.001	1.03	0.98–1.08	0.193
Middle school	1.98	1.90–2.06	<0.001	1.27	1.22–1.32	<0.001
High school	1.85	1.80–1.89	<0.001	1.31	1.28–1.34	<0.001
College or above (Ref.)	-	-		-	-	

Note: Adjusted for sex, age group, income level, and education level. AOR, adjusted odds ratio; CI, confidence interval; Ref., reference category.

**Table 4:** Subgroup analysis of smoking and high-risk drinking among single-person households.

Variables	Smoking AOR (95% CI)				High-Risk Drinking AOR (95% CI)			
	2020 vs. 2019	*p*	2021 vs. 2019	*p*	2020 vs. 2019	*p*	2021 vs. 2019	*p*
Year (reference: 2019)								
Overall	0.98 (0.93–1.04)	0.573	0.99 (0.93–1.05)	0.652	0.87 (0.82–0.93)	<0.001	0.81 (0.76–0.86)	<0.001
Sex								
Male	1.00 (0.94–1.08)	0.898	1.01 (0.95–1.08)	0.702	0.86 (0.80–0.93)	<0.001	0.78 (0.73–0.84)	<0.001
Female	0.90 (0.80–1.01)	0.072	0.88 (0.78–0.98)	0.025	0.86 (0.79–0.93)	<0.001	0.82 (0.73–0.91)	<0.001
Age group								
19–29	0.99 (0.88–1.13)	0.925	0.97 (0.86–1.10)	0.614	0.85 (0.75–0.97)	0.013	0.71 (0.63–0.81)	<0.001
30–44	0.85 (0.76–0.95)	0.005	0.79 (0.71–0.88)	<0.001	0.85 (0.76–0.95)	0.004	0.76 (0.68–0.85)	<0.001
45–64	1.06 (0.97–1.16)	0.188	1.05 (0.97–1.15)	0.245	0.90 (0.82–0.99)	0.023	0.88 (0.80–0.96)	0.006
≥65	0.94 (0.84–1.06)	0.298	1.11 (0.99–1.24)	0.071	0.82 (0.70–0.95)	0.011	0.83 (0.71–0.97)	0.022

Note: Adjusted for sex, age group, income level, and educational level. AOR, adjusted odds ratio; CI, confidence interval. Reference year = 2019.

## Data Availability

The data used in this study are publicly available from the Korea Disease Control and Prevention Agency (KDCA). The raw data of the Korea Community Health Survey (KCHS) can be accessed at https://chs.kdca.go.kr.

## Abbreviations

The following abbreviations are used in this manuscript:

KCHS	Korea Community Health Survey
KDCA	Korea Disease Control and Prevention Agency
AOR	Adjusted odds ratio
CI	Confidence interval
COVID-19	Coronavirus disease 2019
